# PEIS: a novel approach of tumor purity estimation by identifying information sites through integrating signal based on DNA methylation data

**DOI:** 10.1186/s12859-019-3227-1

**Published:** 2019-12-30

**Authors:** Shudong Wang, Lihua Wang, Yuanyuan Zhang, Shanchen Pang, Xinzeng Wang

**Affiliations:** 1 College of Computer Science and Technology, China University of Petroleum (East China), Qingdao, Shandong China; 2 School of Information and Control Engineering, Qingdao University of Technology, Qingdao, Shandong China; 3 College of Mathematics and Systems Science, Shandong University of Science and Technology, Qingdao, Shandong China

**Keywords:** DNA methylation, Differential methylation, Integrating signal, Tumor purity

## Abstract

**Background:**

Tumor purity plays an important role in understanding the pathogenic mechanism of tumors. The purity of tumor samples is highly sensitive to tumor heterogeneity. Due to Intratumoral heterogeneity of genetic and epigenetic data, it is suitable to study the purity of tumors. Among them, there are many purity estimation methods based on copy number variation, gene expression and other data, while few use DNA methylation data and often based on selected information sites. Consequently, how to choose methylation sites as information sites has an important influence on the purity estimation results. At present, the selection of information sites was often based on the differentially methylated sites that only consider the mean signal, without considering other possible signals and the strong correlation among adjacent sites.

**Results:**

Considering integrating multi-signals and strong correlation among adjacent sites, we propose an approach, PEIS, to estimate the purity of tumor samples by selecting informative differential methylation sites. Application to 12 publicly available tumor datasets, it is shown that PEIS provides accurate results in the estimation of tumor purity which has a high consistency with other existing methods. Also, through comparing the results of different information sites selection methods in the evaluation of tumor purity, it shows the PEIS is superior to other methods.

**Conclusions:**

A new method to estimate the purity of tumor samples is proposed. This approach integrates multi-signals of the CpG sites and the correlation between the sites. Experimental analysis shows that this method is in good agreement with other existing methods for estimating tumor purity.

## Background

An important issue in tumor research is that tumor samples during sampling are always mixed with normal cells, which we refer to as “tumor purity”. The understanding of the pathogenic mechanism of tumor has risen from the physical and chemical carcinogenesis in the past to the virus and mutation carcinogenesis later, and finally to the multi-step and multi-factor carcinogenesis. Accurate measurements of tumor purity can reduce the variance caused by other mixed genes in samples and help more effectively target which genes may be closely related to tumor development. In recent years, estimating the “tumor purity” [1–5] of the sample, i.e. the percentage of tumor cells in tumor sample, has received increasing attention. Traditional tumor purity estimation is basically obtained by pathological researchers through image and image analysis. As well as the later technologies based on cell classification, these methods not only cost human resources but also have high costs, which are not suitable for large-scale promotion. Coincidentally, because of the significant genetic and epigenetic differences between tumor cells and normal cells, it is feasible to use available high-throughput data to estimate tumor purity. There are many methods to estimate tumor purity using gene expression [6], copy number variation [7] and single nucleotide polymorphism [8] as predictors, but few are based on methylation differences.

DNA methylation is a common and important mechanism in gene expression regulation, which is involved in cell differentiation and proliferation, tumorigenesis and other important life activities [9, 10]. Changes in normal methylation patterns of the genome are closely related to the occurrence of tumors [11]. Abnormal DNA methylation patterns (including CpG island hyper-methylation and DNA hypo-methylation) and tumorigenesis have been one of the hot topics in the medical field. DNA methylation is found in almost all cancers and occurs before or at an early stage of cancer, so it is expected to be an ideal marker for early diagnosis of cancer. An important problem in tumor research is that the tumor tissues obtained from clinical trials are highly heterogeneous. They are a mixture of tumor cells, adjacent normal cells, stromal and infiltrating immune cells. In high-throughput DNA methylation experiments, the whole tumor sample is processed to extract DNA from all cells and then the methylation levels are profiled. So, these measurements are actually mixed signals from different cell types [12]. If there is no correct interpretation of the sample mix, there will be deviations in the downstream data analysis, such as differential methylation analysis and sample clustering.

Methods of using DNA methylation data to estimate tumor purity have gradually emerged, nowadays. It is not difficult to find that the current methods of using methylation data to estimate tumor purity are mostly based on the selection of information sites. MethylPurify [1] uses EM algorithm to identify information sites and then infer tumor purity; InfiniumPurify [5] identifies information sites by rank-sum test and estimates tumor purity which combined with Gaussian kernel density; PAMES [2] utilizes the methylation levels of dozens of highly cloned specific CpG sites to evaluate the purity of tumor samples. The common feature of these methods is that they need to select the information difference methylation site first and then carry out purity estimation. Currently, methods for identifying differentially methylation sites based on differences in mean methylation levels between tumors and normal samples have been well studied. However, the estimation results of tumor purity by different methods are disparate. Therefore, it is particularly important to select the differentially methylation sites related to tumor as the information sites. Early on, IMA [13] used Wilcoxon rank-sum tests to identify differential methylation sites by comparing mean values. Afterwards, FastDMA [14] used variance analysis based on linear model. Currently, most of the methods which identify differentially methylated sites are based on hypothesis testing, and integrating signal as well as strong correlation among adjacent sites is seldom considered.

**Fig. 1:**
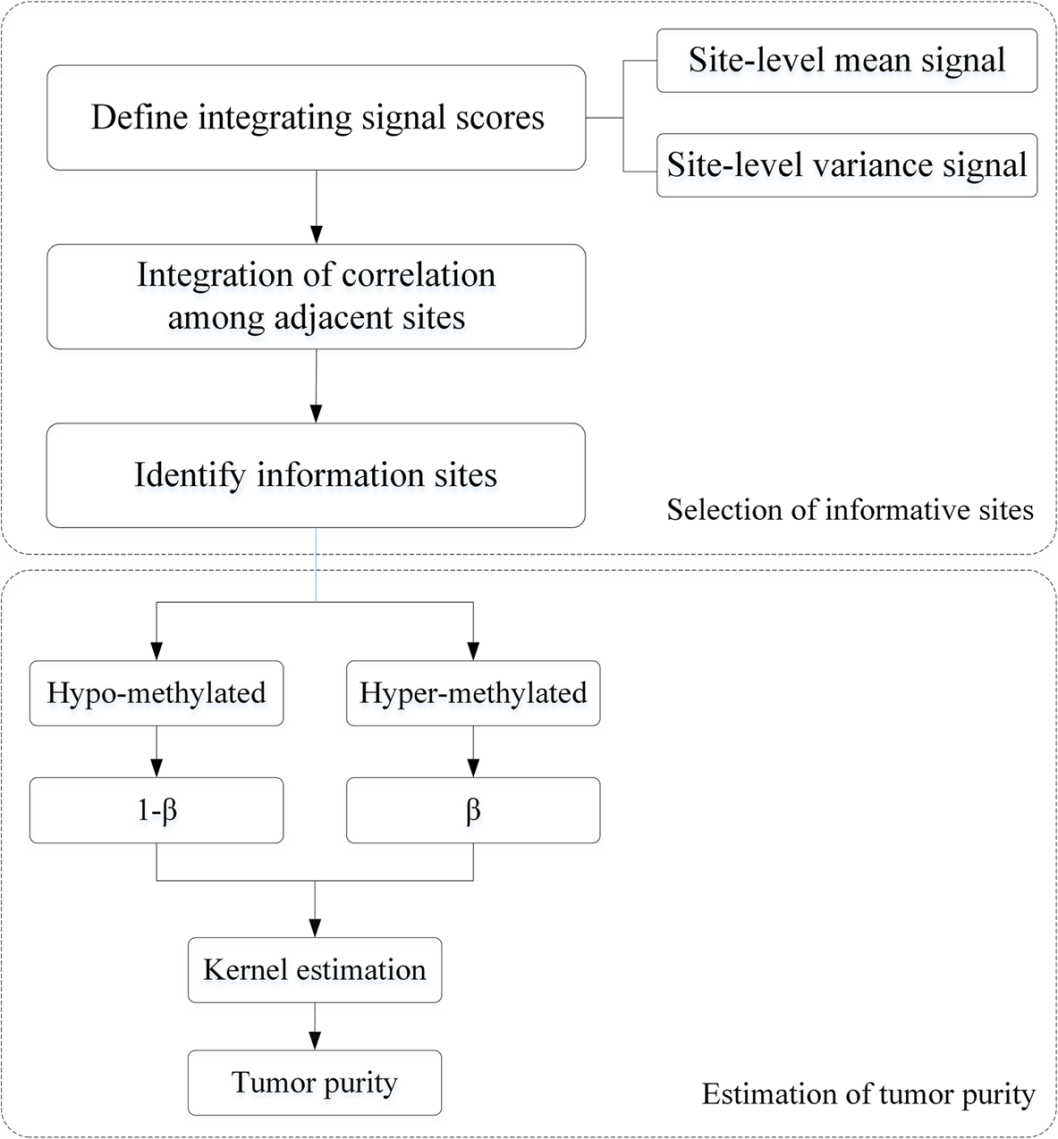
A flowchart to illustrate the PEIS algorithm

Here, we present an approach to estimate tumor purity by identifying information difference methylation sites from the comprehensive signal score of CpG sites, called PEIS (Purity Estimation through Integrating Signal). The method consists of two stages: the first stage selects information sites by integrating signal scores and strong correlation among adjacent sites; in the second stage, the Gaussian kernel density was used to estimate the purity of tumor samples. The algorithm of PEIS is illustrated in Fig. 1. By applying PEIS on more than 600 tumor and normal adjacent samples from 12 tumor types, PEIS shows a high degree of consistency with other existing methods. Also, compared with other methods of selecting information different methylation sites, PEIS has more accurate results in evaluating purity.

## Results

### Experimental data preprocessing

We downloaded DNA methylation data of 12 tumor types of The Cancer Genome Atlas from UCSC Cancer Genome Browser [15]. The tumor types that we used include BLCA (Bladder Urothelial Carcinoma, 21 paired samples), BRCA (Breast invasive carcinoma, 92 paired samples), COAD (Colon adenocarcinoma, 38 paired samples), HNSC (Head and Neck squamous cell carcinoma, 50 paired samples), KIRC (Kidney renal clear cell Carcinoma, 160 paired samples), KIRP (Kidney renal papillary cell carcinoma, 45 paired samples), LIHC (Liver hepatocellular carcinoma, 50 paired samples), LUAD (Lung adenocarcinoma, 29 paired samples), LUSC (Lung squamous cell carcinoma, 41 paired samples), PRAD (Prostate adenocarcinoma, 50 paired samples), THCA (Thyroid carcinoma, 56 paired samples), UCEC (Uterine Corpus Endometrial Carcinoma, 33 paired samples). The number of tumor and normal-adjacent samples of these tumor types is not less than twenty. And all DNA methylation CpG sites were filtered for quality control in advance.

### Tumor purity estimation results from PEIS

**Fig. 2:**
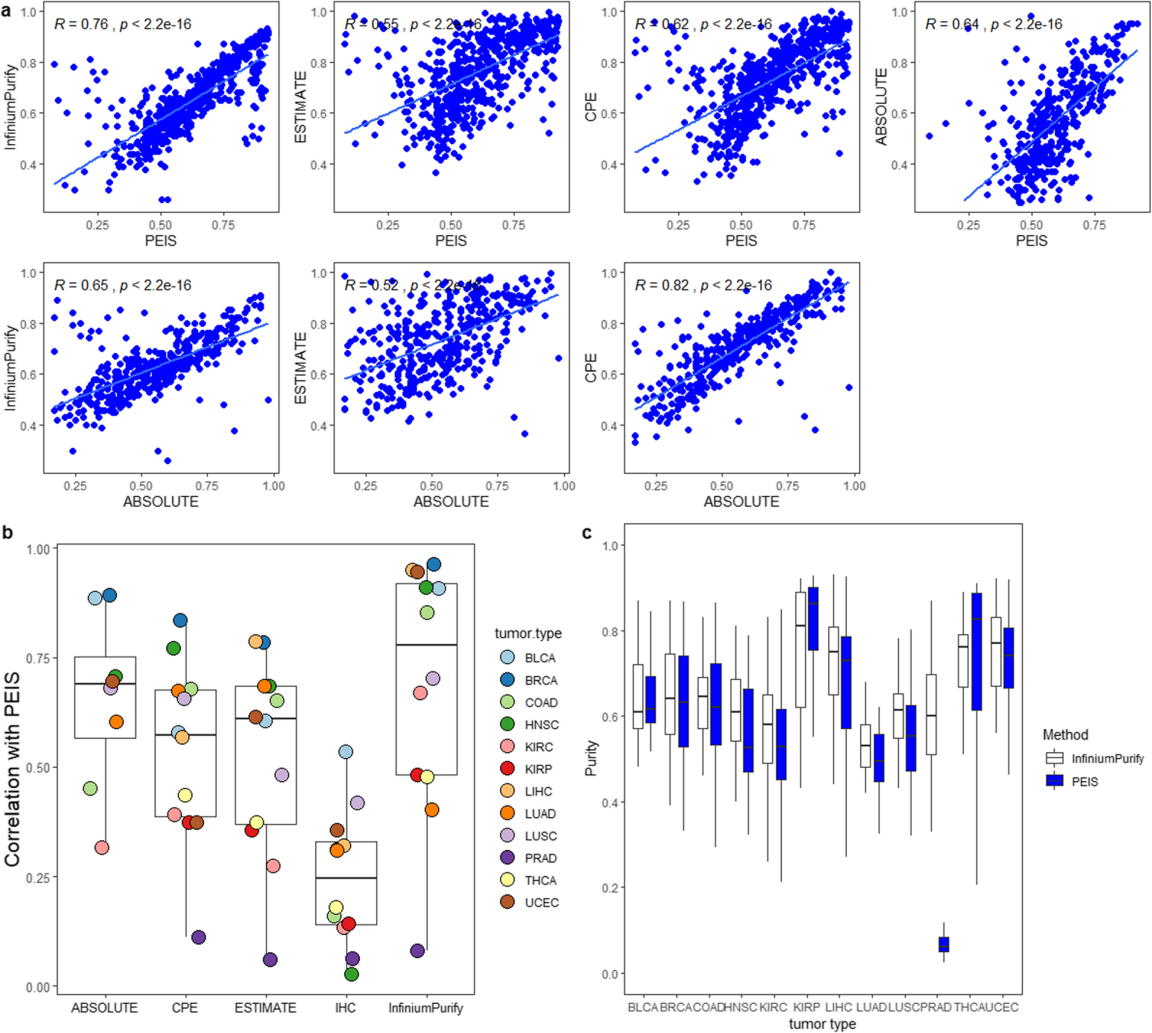
**a** Scatter plots showing comparison of purities for tumor samples. **b** Correlations between PEIS and five kinds of estimates for 12 tumor types. **c** Distribution of tumor purities estimation results from PEIS for 12 tumor types

To illustrate the advantages of PEIS, we applied four methods to our selected and pretreated DNA methylation data (665 samples from 12 tumor types). Figure 2a shows scatter plots of tumor purity comparison for 11 tumor types. ABSOLUTE [16] is called the “gold standard” for estimating tumor purity, and InfiniumPurify is currently the only method to estimate tumor purity using DNA methylation data. The two methods are the focus of our comparison. In general, PEIS is well correlated with other methods. PEIS and InfiniumPurify have the highest Pearson’s correlation (R = 0.76) and the lowest correlation (R = 0.55) with ESTIMATE. But by comparison, PEIS has a higher correlation with each method than ABSOLUTE with them, except for CPE. The estimated result of CPE [16] is the median purity level derived from the normalization of ABSOLUTE, ESTIMATE and other methods. Therefore, this results in a very high correlation between CPE and ABSOLUTE estimation results. For each tumor type, we calculated their Pearson’s correlation, and for most tumor types they have a high correlation (Additional file 1: Figures S1–S4). However, the sample size of each tumor type we used was still relatively small, which is another factor influencing the correlation. Figure 2b shows correlations between PEIS and other methods. Obviously, we can see that the overall correlation between PEIS and IHC is relatively low. It is not ruled out that this is because IHC data source is image, which is not genetic or epigenetic data, and according to relevant studies [16] has shown that IHC is not consistent with other methods. The ABSOLUTE method only has correlation results for eight tumor types, because no other tumor types are currently estimated. For the other three methods, PRAD showed a very low correlation. This can also be seen from Fig. 2c, in the distribution of tumor purity estimation results for 12 tumor types, the purity of PRAD is lower than 0.2.

This may be due to different methylation rates of prostate cancer genes at different stages. Among them, the methylation level of gene Retinoic acid receptor β2 (*RARβ2*) is related to clinical staging [17, 18]. In prostate cancer, the methylation rate of at least two genes in stage D is significantly higher than that in stage B and C. It is possible that as the disease progresses, the number of methylated genes in prostate cancer increases. For one type of tumor, we evaluated the integrating signal score of the overall mean and variance of a CpG site in all samples. Through the study on the information sites of PRAD, we found that the selected information sites showed significant differences in the CpG sites of a single sample, while the remaining samples showed almost no differences. This results in the selected information sites being specific and not applicable to all samples. Therefore, PEIS estimates low purity. Only one of the 50 PRAD tumor samples has a high correlation with other methods, while the rest generally have a low correlation. Based on the study of PRAD clinical data, we find that the initial pathological diagnosis age of this sample is before 45 years old, and the age of the remaining 49 samples is after 50 years old (Additional file 2: Table S1). We do not rule out the possibility that the results of PRAD tumor purity are age-related. Moreover, the data set used in the experiment is processed to remove the locus on the sex chromosomes, while PRAD mainly affect men after the age of 60, and the cancer-related locus may be largely located on the sex chromosomes, which would also affect the subsequent tumor purity estimation results. In addition, we observed that the bottom edge of the THCA boxplot using PEIS is significantly longer than that using InfiniumPurify and some types of tumor show slightly lower consistency, all of these because the number of samples used in our study is small, so even small deviations can reduce the consistency with InfiniumPurify. This is also the part that we will continue to study in depth in the follow-up work.

### Comparison of purity estimation results from different methods

**Fig. 3:**
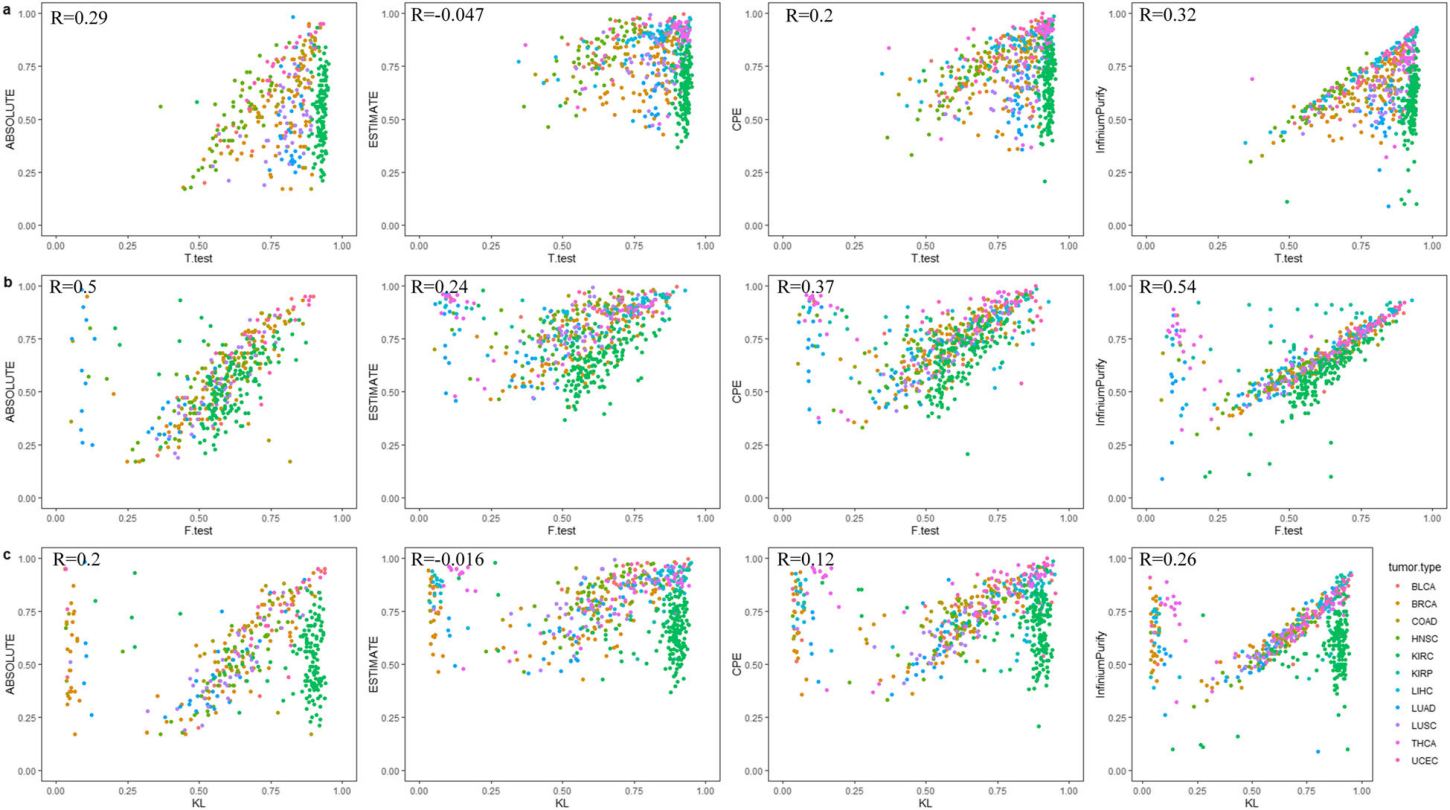
**a** Scatter plots showing comparison of tumor purities from T-test with ABSOLUTE, ESTIMATE, CPE and InfiniumPurify, respectively. **b** Scatter plots showing comparison of tumor purities from F-test with other methods. **c** Scatter plots showing comparison of purities for tumor samples from KL divergence with other methods

The different methylation sites selected by different information sites selection methods are not the same. The selection of tumor-related information sites is crucial for the estimation of tumor purity. The tumor purity estimation results of several different information sites selection methods as shown in Fig. 3. And the different colored sites in the figure represent different tumor types. In general, the results estimated by traditional hypothesis testing methods have relatively low correlation with other methods. The purity estimation results of paired T-test are generally high and close to 1. Paired T-test estimation, which compares the β-value of the overall samples, ignores the influence of individual samples on the whole. As can be seen from Fig. 3a, the purity estimation of almost all samples of KIRC verges on 1. Through some understanding and analysis of the tumor, this may be due to KIRC (Kidney renal clear cell carcinoma) is often clinically mixed with granulosa cell carcinoma and spindle cell carcinoma. It is difficult to distinguish by using the β-value analysis of the whole, so it may affect the estimation of the true purity. However, this reason is only our speculation at present, which needs to be further explored and confirmed. F-test itself is not suitable for selecting information sites, because too many information sites are selected according to the significance of mean difference. Here, the purity estimate obtained is the result of the combination of F-test and T-test to select information sites. It is not difficult to see that the result of F-test combined with T-test is much better than that of T-test alone, as shown in Fig. 3b. The hypothesis test method of KL divergence is not suitable for identifying information sites, because it considers the proportion of β-value of each sample to the whole, and ignores the methylation information carried by the sample itself. Here, the KL divergence method we use is improved by Zhang et al. [19], hereinafter referred to as KL divergence. According to Fig. 3c, it can be seen that some tumor purity estimated by KL divergence is obviously high, while a small part is obviously low. It was found that the purity of KIRC tumor samples estimated by KL divergence was generally higher, but the PRAD estimation results were highly correlated with InfiniumPurify. For PRAD which tumor purity estimation are not ideal by PEIS, the result of KL divergence estimation is in good agreement with other methods. This also indicates that due to tumor heterogeneity, information sites identified by different methods are not the same and different information sites selection methods behave differently for different types of tumor.

## Discussion

Accurate estimation of tumor purity is of great significance for subsequent differential methylation analysis and genetic analysis. Using the available genetic or epigenetic data to estimate tumor purity has more biological value and reference significance. Tumor heterogeneity brings difficulties and opportunities to estimate tumor purity. Currently, gene expression data and copy number variation (CNV) data are mostly used to estimate tumor purity. However, methods for estimating purity using DNA methylation data are far fewer. With these considerations in mind, we propose a method to integrating signal and correlation among adjacent sites to identify information sites related to tumor purity based on DNA methylation data. By applying our method to 12 tumor types, we found that for most tumor samples, our estimates were highly consistent with other commonly used methods.

There are many types of data on cancer studies, such as gene mutations, gene expression and DNA methylation. Nevertheless, none of the data was specifically used to estimate tumor purity, in other words, tumor samples estimation results from these data are not absolutely accurate. Every data has its drawbacks in the estimation process. For example, gene expression data can show huge differences because of slight differences in DNA. The estimation results of copy number variation data are extremely uncertain. Therefore, comprehensive use of CNV and SNV (somatic number variation) to evaluate tumor purity has emerged. By comparison, DNA methylation data are more stable. Of course, the estimation of DNA methylation data is only a supplement to the existing results, which cannot be said to be the standard.

It is worth noting that most methods of estimating tumor purity by DNA methylation are based on the selection of information differential methylation sites. The selection of information sites has a great influence on the estimation results. That is because the selected information sites contain low tumor information. However, this cannot be used as a criterion to evaluate the selection method of differentially methylated sites. Therefore, the key to estimate tumor purity using DNA methylation data is to select information sites containing more tumor information. This is also the direction that we should make continuous efforts in the future. It can also be seen from the experimental results in this paper that the same method has different results for different tumor types, which is caused by tumor heterogeneity.

During the experiment, we also tried to calculate the influence of all the sites within the range of 1000 bps, but the information sites are more likely to be the sites with close genomic distance, and some of the sites themselves are not differential methylation sites, which will affect the subsequent purity estimation. Perhaps applying this approach to CpG islands will yield better results. This is the part we will study in the following experiments.

## Conclusions

Tumor tissue obtained in clinical work is often mixed with non-tumor cells, which play an important role in tumor growth, progression or drug resistance. It is very significant to correctly estimate and adjust the purity of tumors for subsequent differential methylation analysis and genetic analysis. In order to select tumor-related information sites to estimate tumor purity, we present an approach based on integrating multi-signals and strong correlation among adjacent sites. We apply our method to 12 tumor types and show that our method is highly consistent with the results of other commonly used tools. The correlation between sites is taken into account to make the selected information sites more biological.

## Methods

In this section, we detailedly introduce PEIS which considers CpG site’s integrating signal score and correlation among adjacent sites, and then combine with Gaussian kernel density to estimate the tumor purity. The algorithm of PEIS is illustrated in Fig. 1.

### Selection of informative CpG sites

In selecting information sites, we use a two-stage approach. Firstly, the integrating signal scores of the CpG sites are calculated, and secondly, the correlation among adjacent sites is integrated for score correction.

### Step 1: define integrating signal scores

With reference to [20], the integrating signal score of a CpG site *i* could be expressed as

$$ {S}_i=\frac{\left|{T}_{m_i}\right|}{T_{m_i}}\left({\lambda}_i{m}_i+\left(1-{\lambda}_i\right){v}_i\right), $$

where *m*_*i*_ = *ϕ*^−1^(1 − *p*_*mi*_) and *v*_*i*_ = *ϕ*^−1^(1 − *p*_*vi*_). Here, Φ is standard normal quantile function, and *p*_*mi*_ and *p*_*vi*_ are *P*-values from the two-sided paired T-test testing and from the one-sided F-test testing at CpG site *i*, respectively. The sites with mean and variance signal scores of zero and smaller than zero are not considered. Because the *P*-values of these sites are *p*_*mi*_ > 0.5 and *p*_*vi*_ > 0.5. Here, ${T}_{m_i}$ is the T-statistic of the two-sided paired T-test, where $\frac{\left|{T}_{m_i}\right|}{T_{m_i}}$ indicate whether the CpG site *i* is negative sign or positive sign. Positive signals indicate hyper-methylated, whereas negative signals indicate hypo-methylated. Since the mean and variance signals have different scales, we weight the two scores by *λ*_*i*_ and 1 − *λ*_*i*_, respectively. According to [20], the weight of the balanced mean and variance signal score is defined as

$$ {\lambda}_i=\frac{v_i}{m_i+{v}_i}. $$

In general, we are supposed to multiply our respective weights by the corresponding scores, but this will magnify the effect of one of the signals, the mean or the variance, on the final result. For instance, the mean and variance signals of site *i* are 0 and 38 (i.e. *p*_*mi*_ > 0.5 and *p*_*vi*_ = 1.0*e* − 320), and its signal score is 38; the mean and variance signals of site *j* are 14.9 and 14.9 (i.e. *p*_*mi*_ = 1.0*e* − 50 and *p*_*vi*_ = 1.0*e* − 50), and its signal score is 14.9. Obviously, this can seriously affect the evaluation of the site signal score.

### Step 2: integration of correlation among adjacent sites

The significance of the site is not only determined by the integrating signal of the site itself, but also influenced by the adjacent site. For a CpG site *i*, we define its final integrating signal score (i.e. ${f}_{S_i}$) as

$$ {f}_{S_i}={S}_i+{S}_{i_n}^{\prime }. $$

Here, *i*_*n*_ (n = 1, 2) are the two nearest sites to CpG site *i*, and the genomic distance between these two sites and site *i* is less than 1000 bps. ${S}_{i_1}^{\prime }$ and ${S}_{i_2}^{\prime }$ are the influencing scores of CpG site *i*_1_ and *i*_2_ on site *i*. For the CpG site *i*_1_ which is close to CpG site *i*, We define the influencing score as

$$ {S}_{i_n}^{\prime } $$= $$ \upalpha \cdotp {S}_{i_n} $$.

Where, α indicates the influence ratio of site *i*_*n*_ on *i*, and we consider the influencing ratio to be inversely proportional to the genomic distance. We define the influencing ratio as

$$ \upalpha =1-\frac{distance}{1000}, $$

where, *distance* represents the gene distance of the two sites. On the basis of final integrating signal scores, the top 1000 sites with the highest score are selected. The significance of CpG site (*P*-value) is converted into signal score. The higher the signal score, the stronger the significance of the site.

According to the relevant research and experiments, the site with a genomic distance of less than 1000 bps has the most obvious influence on CpG site *i* [20–22], that is to say, the site with a genomic distance of more than 1000 bps has negligible influence on CpG site *i*. In the actual calculation process, we also consider the influence of all sites within 1000 bps of the calculated CpG site *i* on it. However, some sites were selected as information sites due to the influence of surrounding sites, and these sites originally were not differential methylation sites. According to names of 1000 CpG sites, the corresponding original DNA methylation data are selected for subsequent purity estimation.

### Estimation of tumor purity

Here, the method for estimating tumor purity is derived from Zheng’s InfiniumPurify [5, 23]. According to β-values, the selected information sites were divided into hyper-methylated and hypo-methylated sites. The β-value of information sites in tumor samples is transformed as follows: the β-value of hyper-methylated information sites remained unchanged, while that of hypo-methylated information sites were converted to 1-β. These transformation rules are based on whether the β-value is greater than 0.5, which is not affected by other factors. Then the transformed methylation levels of information sites are processed by Gaussian kernel density estimation method. The purity is estimated by density function.

## Supplementary information

**Additional file 1.** Supplementary figures, results and information.

**Additional file 2.** Supplementary table.

## Data Availability

All data used in the experiments are from public databases (UCSC). The datasets generated during the current study are available from the corresponding author on reasonable request.
