# Supplementary material for: PEIS: a novel approach of tumor purity estimation by identifying information sites through integrating signal based on DNA methylation data
Source: BMC Bioinformatics. 2019 Dec 30;20(Suppl 22):714. doi: 10.1186/s12859-019-3227-1 (PMC6936156; doi:10.1186/s12859-019-3227-1)

**Figure S1.** Comparison of tumor purities estimated by PEIS and ABSOLUTE for 12 tumor type.


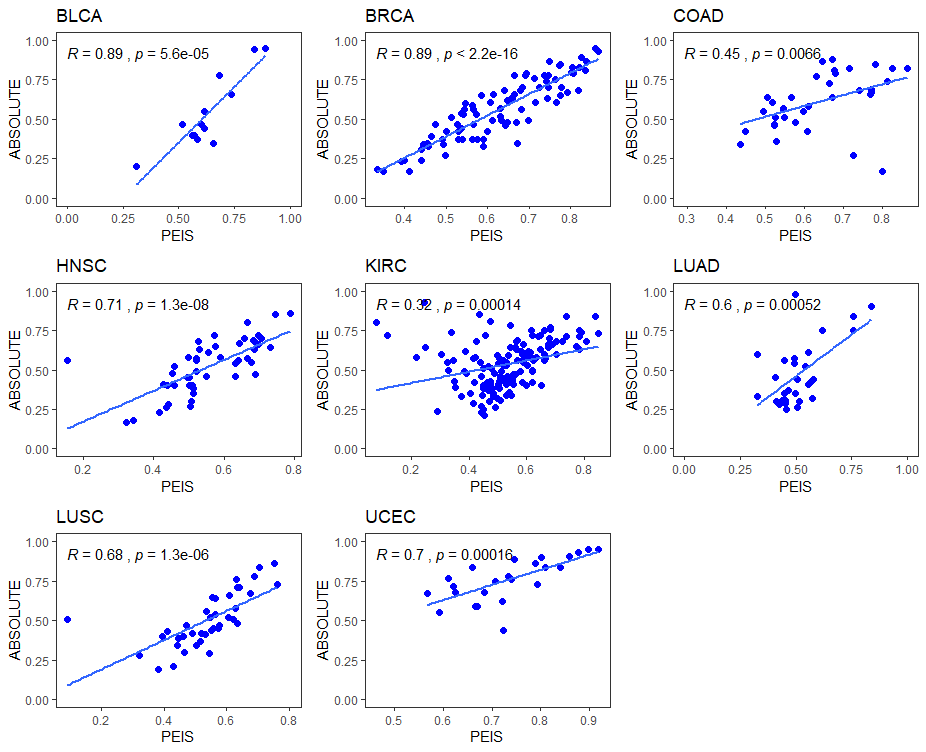


**Figure S2.**Comparison of tumor purities estimated by PEIS and ESTIMATE for 12 tumor type.


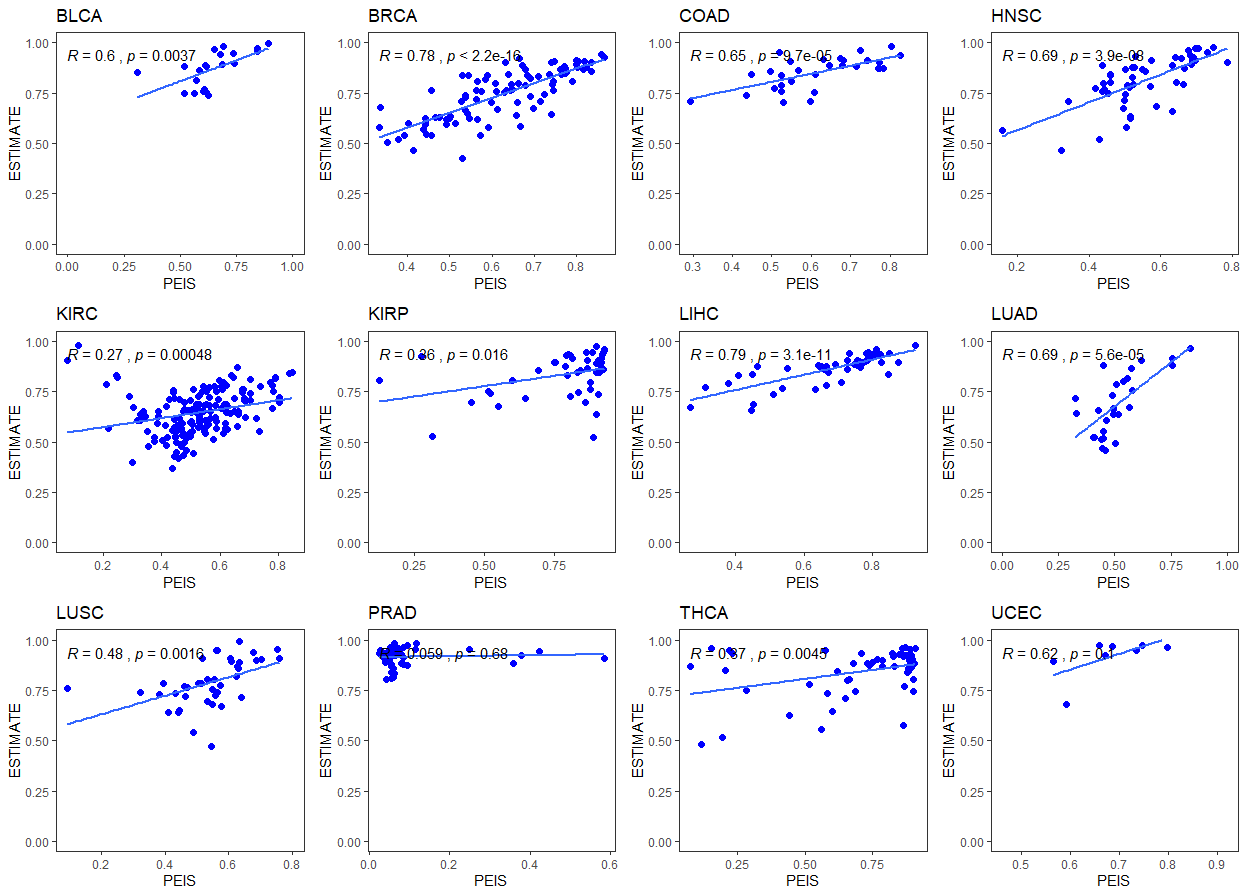


**Figure S3.**Comparison of tumor purities estimated by PEIS and CPE for 12 tumor type.


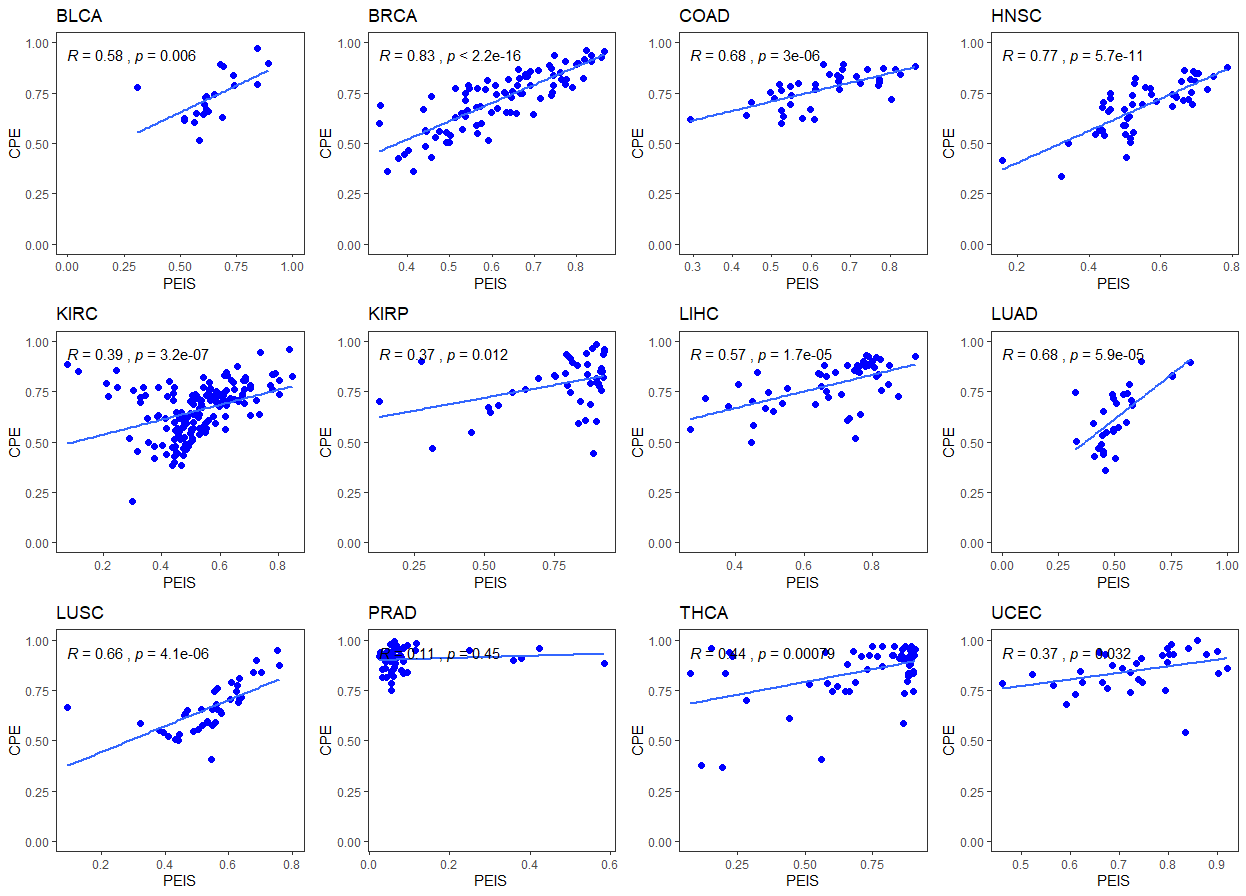


**Figure S4.**Comparison of tumor purities estimated by PEIS and InfiniumPurify for 12 tumor type.


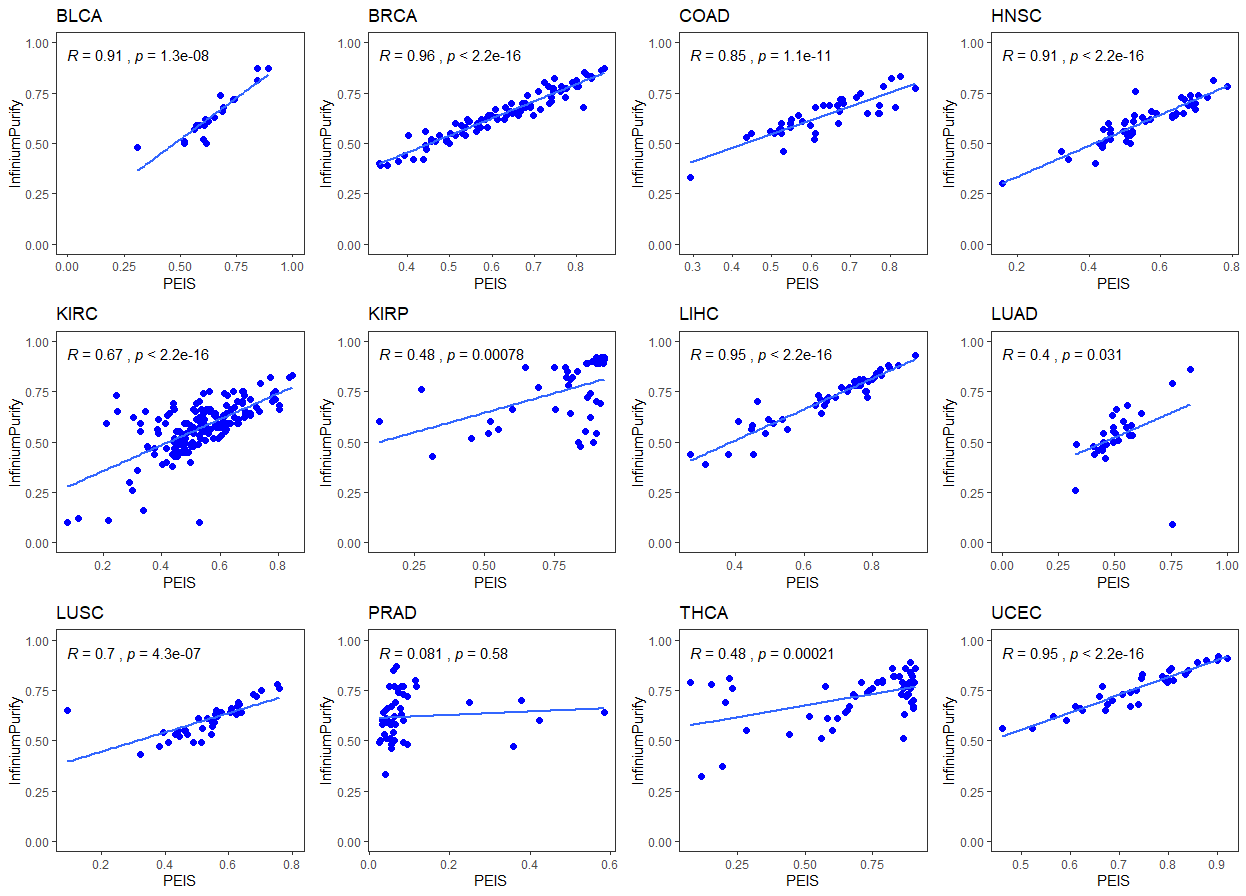

Supplement: Supplementary file 1 — Additional file 1. Supplementary figures, results and information. [file 12859_2019_3227_MOESM1_ESM.docx]
